# An Easy and Certain Method of Performing Catheterism

**Published:** 1845-04

**Authors:** J. G. Maisonneuve


*An easy and certain method of performing Catheterism, even in the most difficult cases.* By J. G. Maisonneuve. In the hands of the ablest and most experienced surgeons, catheterism, in cases of retention of urine, is often a difficult and sometimes a dangerous operation, and, in those of an inexperienced surgeon, it is daily a source of serious accidents. Of late, numerous methods have been proposed to facilitate this operation; and Dr. Maisonneuve proposes the following: Introduce into the urethra a very small gum-elastic bougie, and, when it has reached the bladder, slip over it a catheter, open at both ends. The passage of the latter inwards is facilitated by a bit of silk passed through it and then tied to the extremity of the bougie. To cause the catheter to penetrate easily and without pain into the bladder, it is sufficient to push it onwards on the bougie, drawing gently all the time on the silk. This method has succeeded in all the cases in which the author employed it, some of these being very difficult ones; and, from these facts, he concludes: first, that catheterism, performed in the way just described, is, of all the known methods, the easiest and most certain; second, that it succeeds wherever the other methods are applicable; third, that it succeeds where the others fail; fourth, that it sets aside all painful trials, all ruptures of the canal, all false passages and all the accidents which they give rise to; fifth, that to perform it, no peculiar skill is needed; on the contrary, it may be employed by persons not at all accustomed to such an operation; sixth, that it enables us to set aside the numerous instruments proposed to overcome the different obstacles encountered.—*Ib.*

